# THE ROLE OF FILIAL PIETY IN AGING AND CAREGIVING AMONG CHINESE AND CHINESE AMERICANS

**DOI:** 10.1093/geroni/igae098.3754

**Published:** 2024-12-31

**Authors:** Chunhong Xiao, Rita Jablonski, Patricia Patrician, Aoyjai Montgomery, Youhua Wang, Adelais Markaki

**Affiliations:** University of Alabama at Birmingham/School of Nursing, Birmingham, Alabama, United States; University of Alabama at Birmingham, Birmingham, Alabama, United States; University of Alabama at Birmingham/School of Nursing, Birmingham, Alabama, United States; University of Alabama at Birmingham, Birmingham, Alabama, United States; Southwest University, Chongqing, Chongqing, China (People’s Republic); University of Alabama at Birmingham, Birimingham, Alabama, United States

## Abstract

Understanding filial piety as it pertains to aging and caregiving among Chinese and Chinese Americans (CCAs) is expected to bridge the cultural chasm in health care and improve the quality of rendered services. This concept analysis aims to understand the role of filial piety relevant to caring for aging CCAs and to propose an operational definition. We used four databases and followed Walker and Avant’s method for data extraction and analysis. A total of 26 studies met inclusion criteria and were chosen for synthesis. Synthesis of evidence identified four antecedents: (a) filial obligation as a ‘cultural gene’, (b) sense of altruism, (c) familial solidarity, and (d) societal expectation of ‘birth right’. Attributes included: familial material and emotional support, obedience, pious reverence, and societal norm. Consequences were related to caregiver burden, psychological and physical well-being, quality of life, and health equity. Operational definition of filial piety is: an intrinsic desire of adult children to support parents/elders materially and emotionally as well as an extrinsic desire to adhere to the Chinese societal moral tenet to honor family and be honorable children. Future study should examine dual roles of filial piety as a moderator of role appraisal to caregiver burden and as a mediator of resources. The permeating cultural value of filial piety affects help-seeking behavior, caregiving, family relationships, and caregiver health for CCAs. Understanding the meaning, importance, and effect of filial piety for Asians and CCAs is essential for healthcare providers to provide culturally sensitive social and psychological support to caregivers.

